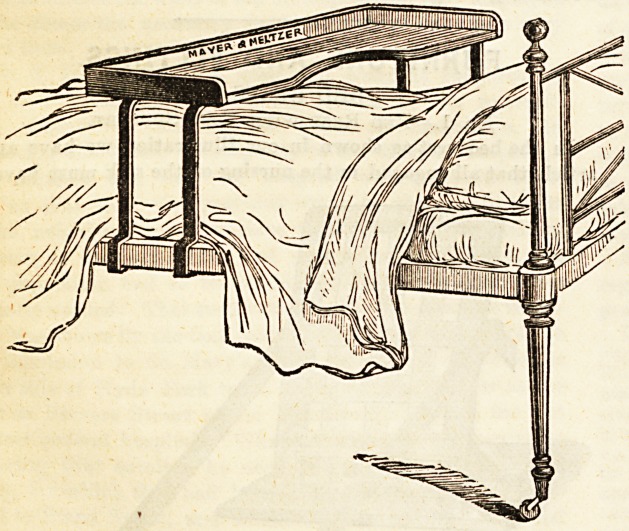# Bed Table

**Published:** 1892-05-28

**Authors:** 


### BED TABLE.

Messrs. Mayer and Metzler supply bed tables, as contrived by Mr. Newton H. Nixon, with a very practical advantage over those usually made entirely of wood. Their tables can be adjusted to the frame of any hospital or infirmary bed or cot being provided with steel legs which give considerable play. The method of adjustment is clearly shown in our illustration.

## Figures and Tables

**Figure f1:**